# Heart regeneration from the whole-organism perspective to single-cell resolution

**DOI:** 10.1038/s41536-024-00378-8

**Published:** 2024-11-15

**Authors:** Xiaoxin Chen, Xiaochen Zhong, Guo N. Huang

**Affiliations:** 1 Cardiovascular Research Institute and Department of Physiology, University of California, San Francisco, San Francisco, CA USA; 2 Eli and Edythe Broad Center of Regeneration Medicine and Stem Cell Research, University of California, San Francisco, San Francisco, CA, USA; 3 Bakar Aging Research Institute, University of California, San Francisco, San Francisco, CA USA

**Keywords:** Cell-cycle exit, Transcriptomics, Translational research, Heart failure

## Abstract

Cardiac regenerative potential in the animal kingdom displays striking divergence across ontogeny and phylogeny. Here we discuss several fundamental questions in heart regeneration and provide both a holistic view of heart regeneration in the organism as a whole, as well as a single-cell perspective on intercellular communication among diverse cardiac cell populations. We hope to provide valuable insights that advance our understanding of organ regeneration and future therapeutic strategies.

## Introduction

Heart failure is a global pandemic affecting millions of people worldwide, and its prevalence is increasing. The adult mammalian heart is notorious for its incapacity to regenerate after ischemia-reperfusion or myocardial infarction injuries, ultimately leading to the development of heart failure. Traditional treatments can only slow the decline of heart function, but most patients eventually progress to heart failure, necessitating heart transplantation. Over the last two decades, various approaches, including transplantation of adult stem cells isolated from skeletal muscle, bone marrow, blood, or fat tissue, have been explored to replenish lost or damaged cardiomyocytes with the aim of restoring cardiac function. Unfortunately, these approaches have demonstrated minimal to modest beneficial effects on heart function recovery, and heart transplantation remains the only definitive cure. Therefore, there is an urgent need to identify effective approaches for heart regeneration.

Cardiomyocytes, or cardiac muscle cells, were originally perceived as *elementi perenni*, or “cells of static cell populations” at the turn of the 19^th^ century^1^. Most scholars at the time believed that cardiomyocytes were akin to skeletal muscle cells, where proliferation and differentiation are mutually exclusive processes. This meant that mature, differentiated heart muscle cells were no longer able to undergo mitosis and cell replication. This dogma largely holds true for cardiomyocytes in mammalian species after a certain point in early postnatal development, when undifferentiated heart muscle cells become nonexistent. However, an exceptional capacity for adult cardiomyocytes to dedifferentiate and replicate has been found in non-mammalian amphibians.

One of the first accounts of cardiac regeneration in adult newt ventricle post-injury was published in 1974 by John and Jean Oberprillar^2^. The repair process involving blood clot formation, lymphocytic cell activity, and connective tissue development was observed. More importantly, myocytes were observed to have undergone DNA synthesis using electron microscopy. A few years later, Nag et al.^3^ found that adult newt cardiomyocytes are capable of mitosis in vitro. Moreover, they observed that mitotic activity was restricted to explanted cardiomyocytes that appeared morphologically dedifferentiated. Thereby, they concluded that the process of reprogramming is integral to adult myocardium regeneration.

Fast forward a few decades to 2002, when Ken Poss discovered in vivo cardiomyocyte regeneration in response to apical resection in zebrafish^4^. Regeneration was not only limited to observable mitotic activity; the lost ventricular wall (up to 20% of the entire ventricle) could also be created. Following his seminal discovery, the field of heart regeneration entered an explosive phase.

Enormous advances in the knowledge of heart regeneration have been made in the last two decades^5^. The recent emergence of single-cell omics technologies, particularly transcriptomics, has transformed our approach to studying organs and organisms, enabling an unparalleled precision in comprehending cell populations^6^. Single-cell transcriptomics facilitates insights into the prevalence and variability of gene expression, as well as the co-expression patterns of genes at the individual cellular level, promoting a cell centered perspective. This method entails identifying new cellular markers and transcriptional patterns to distinguish between cell types and cell states with remarkable accuracy^7–9^. Examining gene sets enriched in specific cell types or states enables deductions about intracellular regulatory networks and intercellular pathways across distinct cell populations, particularly focusing on genes encoding ligand-receptor pairs^10,11^. Such analyses illuminate the mechanisms underlying coordinated cellular communication, which might be obscured in bulk analysis.

In this review, we discuss the fundamental questions related to the divergent cardiac regenerative potential across phylogeny and ontogeny. Furthermore, we propose considering heart regeneration from the perspectives of the external environment, the whole-organism system, and cellular crosstalk. Firstly, we will take a bird’s-eye view of heart regeneration by considering it beyond the organism. The environment in which the organism dwells also plays a role; factors such as oxygen levels and the food we eat might all play important roles. Secondly, we will consider the organism as a system, with the heart as an essential organ closely interconnected with the rest of the body. Heart regeneration is influenced by factors such as hormones (circulating factors), the immune system, and the nervous system. Lastly, we will consider heart regeneration as a local response to tissue injury or loss, controlled by a complex network of cellular and molecular mechanisms. We will explore the contributions of various cardiac cell populations during cardiac repair and regeneration. This is made possible by recent advances in techniques that enable single-cell resolution analysis of the heart, including single-cell RNA sequencing (scRNA-seq), single-nucleus RNA sequencing (snRNA-seq), single-cell assay for transposase-accessible chromatin with sequencing (scATAC-seq), and spatial transcriptomics, which provide insights at the cellular and sub-cellular levels.

## Questions about the divergent cardiac regenerative potential across phylogeny and ontogeny

### Is cardiac regeneration an ancestral trait or evolutionary novelty?

From planarians and acoels, which can undergo whole-body regeneration when the head or tail end is removed, to salamanders, which can regenerate a limb if lost to prey, regeneration is an umbrella term that manifests differently across various species in the animal kingdom. Jonathan MW Slack offered a tentative proposal that whole-body regeneration is likely an ancestral trait^12^. Likewise, current evidence also suggests that cardiac regeneration is more likely an ancestral character of vertebrates rather than an evolutionary novelty. In almost all vertebrate species, the heart can regenerate during embryonic and neonatal stages. Adult heart regeneration is also well documented in some fish, amphibian, and reptile species although these animals must retain a significant portion of functional heart tissue after injury to survive. In contrast, some invertebrate species can live without a heart and more remarkably, can regenerate a new heart de novo from body parts that initially lacks one. For example, earthworms can regrow an amputated anterior portion of their body, including the heart and brain, within 5–10 days^13^. Recently, Mitoh and Yusa reported that sacogglossan sea slugs *Elysia* cf. *marginata* and *E. atroviridis* can regenerate their hearts within seven days after decapitation and their posterior body within three weeks^14^. With the expansion of species tested for cardiac regenerative capacity, we foresee a clearer picture of the phylogenetic distribution of heart regeneration and a better answer to this fundamental question.

### Is heart regeneration a neutral trait?

It is difficult to imagine that robust cardiac regenerative potential confers amphibians and fish survival advantages in evolution since if their hearts were damaged by predators, half of the body would be gone, and the animal is unlikely to survive such major trauma. Interestingly, among teleost fish, certain species such as killifish, goldfish, and zebrafish are well documented for their ability to regenerate cardiomyocytes. However, such abilities are lost in other species like the medaka and Pachón cavefish, which form a fibrotic scar post-cardiac injury, similar to the adult human heart^15^. As outlined by Marshall and colleagues, certain frog species, such as the *Xenopus tropicalis* have cardiac regenerative potential, whereas its close cousin, *Xenopus Laevis*, does not^16^. These examples showcase that such regenerative potential does not significantly impact the survival of vertebrate species and likely remained a neutral trait throughout most of animal evolution.

### Why is cardiac regenerative potential lost in adult humans?

For most of the time since Homo sapiens have existed, our life expectancy has been less than 30 years^17^. However, in just the last one and a half centuries, life expectancy has risen to almost 80 years^16^. Worldwide, the mean age of onset for coronary artery disease is well over 50 years^18^. Therefore, for thousands of years prior to industrialization and modernization, there was no selection pressure during most of human evolution to retain cardiac regenerative potential. To explain why adult humans lose cardiac regenerative potential, several models have been proposed, including an oxygen-rich environment, the development of endothermy, a complex adaptive immune system, and cancer risk trade-offs^19^.

**Fig. 1:**
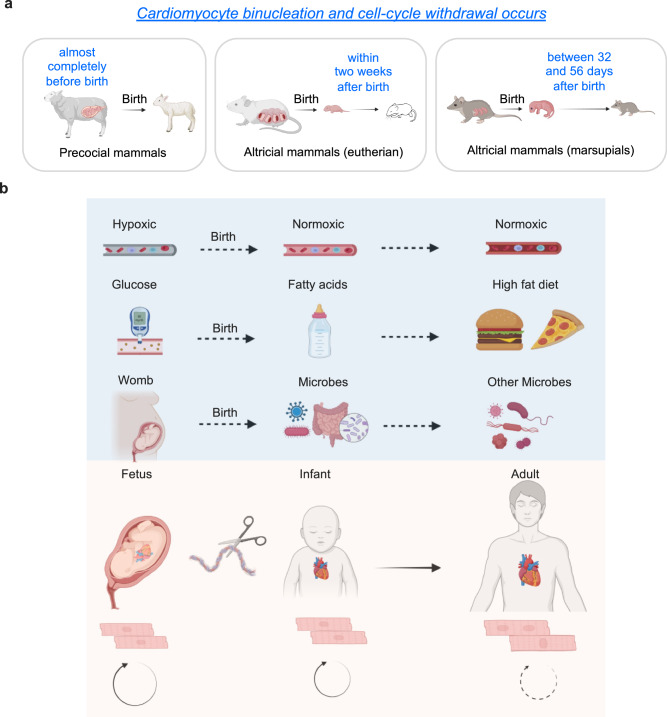
Evidence for internal and external factors inducing cardiomyocyte binucleation and cell-cycle arrest, key barriers for heart regeneration potential. **a** Cardiomyocyte binucleation and cell-cycle arrest occurs at different perinatal windows in different mammalian species. **b** External factors affecting cardiomyocyte proliferation and binucleation. The switch from hypoxic environment to normoxic condition after birth. The transition to fat-rich diets happens after birth. The changes from relative-sterile womb to microbiome exposure after birth. These changes of external factors coincide with postnatal cell cycle arrest and cardiomyocyte maturation in certain mammalian species. These images were created with Biorender.com.

Like almost all other mammalian species characterized so far, humans cannot undergo cardiomyocyte regeneration in adulthood. During development, most mammalian species like mice, rats, and pigs are capable of cardiomyocyte proliferation and heart regeneration, but they lose these abilities one week after birth^20^. These findings suggest that the loss of cardiac regenerative potential in postnatal mammals is under certain selection pressure associate with the postnatal oxygen-rich environment and fat-rich diet. However, it is important to note that several studies have questioned whether postnatal external factors are primary drivers of cardiomyocyte cell cycle withdrawal, the major barrier for heart regeneration (Fig. 1a). One study showed that in precocious mammals, such as lambs, almost all cardiomyocytes exit the cell cycle and become polyploid before birth^21^. Another study found that opossum cardiomyocytes retain cell cycle activity for five weeks postnatally^22^. Thus, both studies suggest that postnatal factors may play a modulatory rather than a determinant role in restricting cardiomyocyte renewal and heart regeneration.

Here, we review crucial molecular and cellular regulators of heart regeneration. In the molecular section, we will first discuss how external factors like oxygen levels, diet, and microorganisms modulate cardiomyocyte proliferation capacity (Fig. 1b), and later summarize reported internal systemic drivers of cardiomyocyte cell cycle arrest and polyploidization as well as loss of heart regenerative potential. In the cellular section, we will emphasize recent progresses from scRNA-seq, ATAC-seq, and spatial transcriptomics analyses.

## Heart regeneration in the organism as a whole

### External factors

#### Oxygen

High ambient oxygen levels might inhibit heart regeneration. One factor common to many organisms capable of heart regeneration is their oxygenation state. Birth significantly transitions an infant from a relatively hypoxic intrauterine environment to a normoxic one. Newborn rats, mice, pigs, and even humans can inherently possess the ability to regenerate damaged heart tissues, but this capacity is lost within the first week after birth in mice and pigs^23–26^. Research by Puente et al.^27^ suggests that transition to an oxygen-rich postnatal environment serves as an upstream signal that leads to the cell cycle arrest of cardiomyocytes. Postnatal hypoxemia, scavenging of reactive oxygen species, and inhibition of DNA damage response all extend the postnatal proliferative window of cardiomyocytes, whereas hyperoxemia and generators of reactive oxygen species generators shorten it. Consistently, in adult mice, gradually decreasing inspired oxygen by 1% and maintaining it at 7% for two weeks leads to the inhibition of oxidative metabolism, reduced production of reactive oxygen species, decreased oxidative DNA damage, and reactivation of cardiomyocyte mitosis^28^. Notably, exposure to hypoxaemia one week after the induction of myocardial infarction has been shown in certain studies to induce a robust regenerative response, characterized by decreased myocardial fibrosis and improved left ventricular systolic function^28^.

However, a more recent study has produced conflicting results, showing that systemic hypoxia only induced proliferation in the right ventricle, not the left ventricle^29^. Therefore, whether oxygen levels regulate cardiomyocyte proliferation and heart regeneration in the left ventricle, which is most relevant to patients after a heart attack, awaits more investigations.

### Nutrition and diet

The perinatal period is characterized by dramatic changes in nutrition. In utero, the fetus receives a continuous supply of low-fat and high-carbohydrate substrates through placenta. After birth, the newborn is fed a high-fat, low-carbohydrate diet^30^. These nutrition changes shift cardiomyocyte fuel preference from glucose to fatty acids for postnatal energy production^31,32^. Coincidentally, the postnatal maturation of cardiomyocytes is also characterized by a metabolic switch from glycolysis to fatty acid oxidation. Therefore, it is tempting to speculate that the high-fat milk contains molecules that orchestrate cardiomyocyte maturation, and that manipulating the nutrition supply or inhibiting fatty acid oxidation could potentially revert cardiomyocytes to a more immature, regenerative state. In line with this idea, it was recently reported that γ-linolenic acid in maternal milk drives cardiac metabolic maturation^33^. Neonatal mice fed fat-deficient milk showed an extended postnatal cardiomyocyte proliferative window^34^. Carnitine palmitoyltransferase (Cpt) are mitochondrial integral membrane enzymes that are critical for the import of acyl-CoA into the mitochondrial matrix and the beta oxidation of long-chain fatty acids. Genetic inactivation of *Cpt1b* abrogates fatty acid oxidation in cardiomyocytes, enhances resistance to hypoxia, and stimulates cardiomyocyte proliferation, facilitating heart regeneration after ischemia-reperfusion injury^35^. Moreover, a heterozygotic loss of *Cpt2* has been previously shown to delay the postnatal cell cycle withdrawal of mouse cardiomyocytes^36^, supporting the role of the fat-rich diet and fatty-acid metabolism in limiting cardiomyocyte proliferation and regeneration.

### Microbes

Upon birth, the infant transitions from a relatively sterile environment in the womb to encountering a multitude of microbes. The primary encounter with bacteria occurs during the journey through the birth canal, followed by immediate exposure through oral, skin, and respiratory contact with the external environment^37^. Newborns possess immature immune systems characterized by underdeveloped neutrophils, monocytes, macrophages, and B and T cells, which exhibit a diminished response to various stimuli^38^. Numerous bacteria that establish residence in the gut and other mucosal surfaces profoundly influence the maturation of the immune system^39^. The immune system gradually develops over the first few months of life in response to environmental pathogen exposures and changes in the gut microbiome^40^. As the child grows, the immune repertoire is further shaped by intercurrent infections and vaccinations^38^. The less mature immune system observed in neonates enables a more balanced regenerative reaction within the mammalian heart through activating cardiomyocyte proliferation and angiogenesis via interleukin-6/signal transducer and activator of transcription signaling^41,42^. Conversely, the proinflammatory and profibrotic reactions in adult mammals contribute to scar formation and hinder cardiomyocyte proliferation^43,44^. One example in support of this idea was shown in a recent study that uncovered a critical role of gut microbiota-derived short-chain fatty acids, including acetate, butyrate and proprionte, in monocyte recruitment and cardioprotection after myocardial infarction^45^.

## Internal Factors

**Fig. 2:**
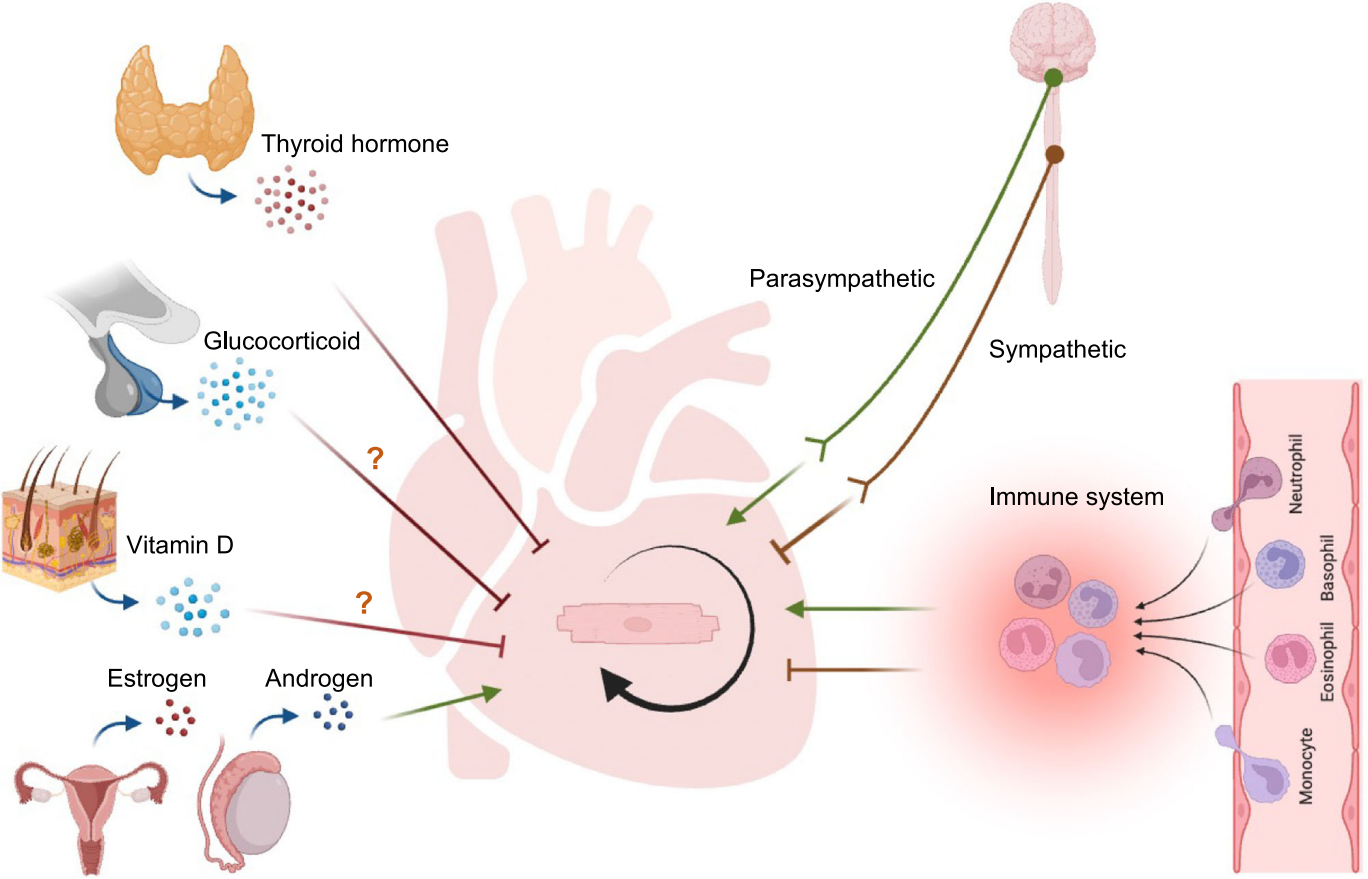
Internal endocrine, neural, and immune regulators of cardiomyocyte proliferation and heart regeneration. Endocrine factors thyroid hormone, glucocorticoid, and vitamin D may inhibit cardiomyogenesis. Sex hormones like estrogens and androgen may promote cardiomyogenesis. Neural system like sympathetic nervous system inhibits cardiomyocyte expansion whereas those from the parasympathetic nervous system promote proliferation. Immune cells modulate cardiac repair and regeneration. The image was created with Biorender.com.

The whole body is an integral system. The regeneration of the heart is no exception and is intrinsically linked to the endocrine, immune system, and nervous system (Fig. 2).

### Endocrine system

Hormones are signaling molecules synthesized and released by various glands to carry messages through the circulatory system to act on distant organs, skin, muscles, and other tissues in the body. Some hormones belonging to amino-acid-derived, steroid, eicosanoid, and protein subclasses have been suggested to be involved in cardiomyocyte proliferation and heart regeneration^46,47^.

### Thyroid hormones

Thyroid hormone signaling is generally considered to be a negative regulator of cardiomyocyte proliferation. Thyroid hormone, produced and released from thyroid glands, is the hormone that’s mainly responsible for controlling the speed of metabolism. Circulating thyroid hormone levels increase more than 50-fold in postnatal mice when thermogenesis increases and animals become endothermic^48^. Mice with cardiomyocyte-specific inactivation of thyroid hormone signaling have more diploid cardiomyocytes, polyploidization, show delayed cardiomyocyte cell cycle exit, and retain cardiac regenerative potential in adults^36^. Conversely, exogenous thyroid hormones inhibit heart regeneration in adult zebrafish, which have low levels of circulating thyroid hormones^36^. Recently, elevation of thyroid hormone levels in neonatal mice was also shown to block cardiac regenerative potential, at least in part by conversion of monocytes and macrophages towards more pro-inflammatory phenotypes^49^. Moreover, the percentage of diploid cardiomyocytes, a proxy of cardiac regenerative potential, across 41 vertebrate species showed inverse correlations with the animal standard metabolic rate, body temperature and serum thyroid hormone level^36^. Altogether, these findings support that loss of heart regenerative capacity in adult mammals is triggered by increasing thyroid hormones and may be a trade-off for the acquisition of endothermy^36^.

### Vitamin D

Vitamin D signaling is usually considered to inhibit cell proliferation in mammals. Vitamin D is mostly produced in the skin and absorbed from food consumed. The liver and kidneys convert vitamin D into active form, which then participates in various physiological processes. Vitamin D has been shown to exert different effects on cardiomyocyte proliferation across phylogeny, with mammals generally experiencing anti-proliferative activities^50,51^, and lower vertebrates undergoing increases in cardiomyocyte proliferation (Table 1)^52^. Surprisingly, cardiac-specific vitamin D receptor knockout mice show no alteration in cardiomyocyte proliferation at postnatal day 14, as indicated by insignificant changes in the percentage of mononuclear cardiomyocytes, as well as mitotic and cytokinetic cardiomyocyte activity compared to controls^51^. Furthermore, vitamin D receptor target genes are not activated during the first two week after birth in mice when most cardiomyocytes withdraw from the cell cycle^51^. These findings suggest that exogenous vitamin D is sufficient to inhibit cardiomyocyte proliferation, but the endogenous signaling pathway mediated by vitamin D receptor is not necessary for neonatal cardiomyocyte cell cycle exit in vivo.

**Table 1:** Studies on vitamin D and glucocorticoids show differing effects on cardiomyocyte proliferation and heart regeneration

	Vitamin D	Glucocorticoids
Mammals	Negative effect: Vitamin D treatment inhibited neonatal rodent cardiomyocyte proliferation in vitro^50,51^ No effect: Cardiac-specific vitamin D receptor knockout mice did not show any effect on cardiomyocyte proliferation at postnatal day 14 in vivo^51^	Negative effect: Dexamethasone treatment inhibited neonatal rodent cardiomyocyte proliferation in vitro^51^. Glucocorticoid receptor ablation in mice after myocardial infarction led to cardiac muscle regeneration in vivo^57^ No effect: Cardiac-specific glucocorticoid receptor knockout mice did not alter cardiomyocyte proliferative activity at postnatal day 14 in vivo^51^
Zebrafish	Positive effect: Vitamin D analog enhanced zebrafish cardiomyocyte proliferation and heart regeneration while vitamin D receptor blockade inhibited regeneration^52^	Negative effect: Dexamethasone /adrenaline^55^ and beclomethasone^56^ led to heart regeneration defects in zebrafish

The factors accounting for the differential responses between zebrafish and mammalian hearts to vitamin D remain unclear. However, it is well known that vitamin D receptor transcriptional targets are species- and context dependent^53^. For example, treatment with a vitamin D receptor agonist in zebrafish has been shown to induce the expression cell cycle genes such as *cyclin*s, *cdk*s, *e2f*s^52^, while activation of the vitamin D pathway in certain mammalian cancer cells led to upregulation of cell cycle inhibitors p27 and p21, as well as downregulation of cyclin D1 and cyclin D3^54^. Thus, the opposite outcomes in cell cycle gene control could explain why vitamin D stimulates cardiomyocyte proliferation in zebrafish while inhibiting cardiomyocyte renewal in mammals.

### Glucocorticoids

Glucocorticoids are generally regarded as inhibitors of cardiomyocyte proliferation. Glucocorticoids are synthesized and secreted by adrenal glands in vertebrates in response to external stressors. It was reported that intermittent crowding triggered an increase in cortisol secretion and blocked the replacement of fibrotic tissue with new myocardium in zebrafish. Simulation of stress through pulse treatment with dexamethasone/adrenaline reproduced the regeneration failure, while inhibition of the stress response with anxiolytic drugs partially rescued the regenerative process^55^. Moreover, treatment with the glucocorticoid beclomethasone also impaired heart regeneration in adult zebrafish^56^.

However, research in mammals has produced conflicting results regarding the physiological role of glucocorticoids in triggering the loss of cardiomyocyte renewal potential (Table 1). One study showed that while glucocorticoid treatment suppresses neonatal mice cardiomyocyte proliferation in vitro, the absence of such signaling does not alter the expression of glucocorticoid target genes or maintain cardiomyocyte proliferative potential in neonatal mice. This suggests that this pathway is not active in neonatal mouse hearts and is dispensable for cardiomyocyte cell cycle exit^51^. In contrast, another study showed that cardiomyocyte-specific glucocorticoid receptor ablation in mice delayed postnatal cardiomyocyte cell cycle exit, hypertrophic growth, and cytoarchitectural maturation^57^. Glucocorticoid receptor ablation or transient pharmacological inhibition after myocardial infarction in juvenile and/or adult mice facilitated cardiomyocyte survival, cell cycle re-entry, and division, leading to cardiac muscle regeneration and reduced scar formation^57^. These contradictory findings highlight the need for further investigation to define the physiological role of glucocorticoid signaling in mammalian cardiomyocyte function and regeneration.

### Sex hormones

Estrogens, androgens, and progestins all belong to sex steroid hormones. Studies using lower vertebrate models suggest that sexual dimorphism reflects a dimorphic cardiac regenerative response. It has been reported that female zebrafish have more cycling cardiomyocytes in both uninjured and cryoinjured regenerating hearts compared to males. Furthermore, exposure to estrogen accelerates heart regeneration in male zebrafish by enhancing cardiomyocyte dedifferentiation and proliferation. Instead, exposure to tamoxifen, an estrogen receptor antagonist, delays heart regeneration in females^58^. On the other hand, progesterone supplementation has been suggested to increase cardiomyocyte proliferation and heart regeneration after myocardial infarction in a progesterone receptor-dependent manner, by increasing YAP expression and signaling^59^.

#### Immune system

The immune response is essential for heart regeneration. On the one hand, immune cell infiltration is required to clear necrotic cells, initiate angiogenesis, and induce fibroblast growth. On the other hand, a rapid resolution and avoidance of autoreactivity is necessary for preventing excessive damage^60^. Therefore, fine tuning of the immune response toward the regenerative immune program might represent a new avenue for heart regeneration studies. Both innate and adaptive immune responses play important roles in heart regeneration. This review mainly discusses the role of immune responses and cardiomyocyte proliferation under the setting of heart regeneration. Comprehensive review of immunomodulation for heart regeneration is outside the scope of this review and can be found elsewhere^61^.

Current evidence suggests that the initiation of acute inflammation is critical in stimulating heart regeneration. Treatment of zebrafish with zymosan or lipopolysaccharides, targeting Toll like receptor 2 and 4, respectively, has been shown to precondition cardiomyocytes, enhancing cell cycle re-entry and cell survival following injury^62^. Medaka, another freshwater teleost, exhibits a weaker immune response to heart injury and lacks both revascularization and cardiomyocyte proliferation compared to zebrafish^63,64^. Consistently, promotion of macrophage recruitment by intraperitoneal injection of the Toll like receptor 3 agonist Poly(I:C) to medaka stimulates revascularization and cardiomyocyte proliferation after injury^65^. In mammals, acute inflammation induced by intramyocardial injection of immunogenic zymosan A particles stimulates cardiomyocyte proliferation in neonatal mice, while cardiac injury-induced regenerative response was suspended after immunosuppression^41^.

#### Nervous system

Autonomic nervous system consists of sympathetic nervous system and parasympathetic nervous system. Sympathetic and parasympathetic neurons exert antagonistic effects on the heart. Sympathetic innervation increases heart rate and contraction force, while parasympathetic innervation does the opposite^66^. Recent studies suggest that the autonomic nervous system controls cardiomyocyte proliferation, indicating that neuronal innervation plays a role in regulating heart regeneration^67^.

### Sympathetic nervous system

The neurons of the sympathetic nervous system are typically synapsed to effector tissues using epinephrine or norepinephrine as neurotransmitters. These neurotransmitters act on α- or β-adrenergic receptors, which are typically G-protein coupled receptors^68^.

Four groups investigated the role of adrenergic receptors in cardiomyocyte proliferation and regeneration independently. First, Tampakakis et al.^69^ showed that inhibition of sympathetic innervation resulted in heart enlargement with an increase in cardiomyocyte number in neonatal mice. Transcriptomic and protein analysis showed down-regulation of the two clock gene homologs Period1/Period2 accompanied by up-regulation of cell cycle genes. Conversely, increasing sympathetic activity by norepinephrine treatment induced Period1/Period2 and suppressed cardiomyocyte proliferation. Furthermore, the two clock genes were found to negatively regulate cardiomyocyte mitosis entry through the Wee1 kinase pathway. Second, Sakabe et al.^70^ found that β1 adrenergic receptor blocker metoprolol enhanced postnatal cardiomyocyte proliferation and extended cardiac regeneration window of postnatal day 7 mice after myocardial infarction, resulting in reduced scar formation and improved cardiac function. The increased cardiomyocyte proliferation was also induced by the genetic deletion of *Gnas*, the gene encoding G protein alpha subunit (Gαs), a downstream effector of β-adrenergic receptor. Genome wide transcriptome analysis revealed that the Hippo-effector YAP, which is associated with immature cardiomyocyte proliferation, was upregulated in the cardiomyocytes of β-blocker treated and *Gnas* cardiac-specific knockout hearts. Moreover, the increased YAP activity is modulated by RhoA signaling. Third, Liu et al. reported that administration of the β-blocker propranolol increased cardiomyocyte division in neonatal mice through YAP-Ect2 signaling axis, which increased the endowment and conferred benefit after myocardial infarction in adults^71^. Last, Payuma et al. demonstrated that inhibition of β-adrenergic receptor alone had mild effects in the control of cardiomyocyte proliferation and binucleation. However, combined treatment with chemical blockers of α-, β-adrenergic receptor, along with thyroid hormone signaling, resulted in profound effects in postnatal mice, including 60% diploid cardiomyocytes, a 60-fold increase in cardiomyocyte proliferation after injury, robust cardiac functional recovery, and minimal scar formation^72^. In sum, these findings support the role of sympathetic nerve and adrenergic receptor activity in regulating cardiomyocyte renewal during heart growth and regeneration, despite differing views on the degree of functional contribution and key downstream genes among these four groups.

### Parasympathetic nervous system

The vagus nerve, part of the parasympathetic nervous system, relays acetylcholine directly to the heart^73^. In zebrafish, Mahmoud et al.^74^ found that hypo-innervation of the adult heart, achieved through myocardial overexpression of *semaphorin3aa*, a known inhibitor of cardiac innervation, reduces cardiomyocyte proliferation and heart regeneration. Disruption of cholinergic signaling with non-selective muscarinic receptor antagonist atropine diminishes cardiomyocyte cell cycle activity and inhibits heart regeneration following apex amputation of the heart. Interestingly, atropine treatment without injury had no impact on zebrafish well-being or on the heart morphology and baseline myocyte proliferation. In mammals, the same group of authors^74^ also demonstrated that both chemical inhibition of cholinergic nerves and mechanical denervation by ablating the left vagus nerve inhibit cardiomyocyte proliferation and impair heart regeneration in neonatal mice following apical resection and myocardial infarction. Reduced expression of cell cycle genes, as well as the growth factors Nrg1 and Ngf, in neonatal mice following cholinergic nerve inhibition suggests that the loss of cholinergic signaling reduces cardiomyocyte proliferation. Moreover, injection of recombinant NRG1 and NGF partially rescued the regenerative response following cholinergic denervation. White et al.^75^ also demonstrated the importance of innervation for regeneration through lineage tracing using Wnt1-Cre-tdTom to identify neural crest derivatives in the developing mouse embryo. Neural crest-derived subepicardial autonomic nerves, which innervate the ventricles, showed robust regrowth and reinnervation during the regeneration of resected ventricular tissue in postnatal day 2 mice. However, this re-growth was inhibited when these nerves were specifically targeted using 6-hydroxydopamine to induce chemical sympathectomy.

## Heart regeneration at single-cell resolution

**Fig. 3:**
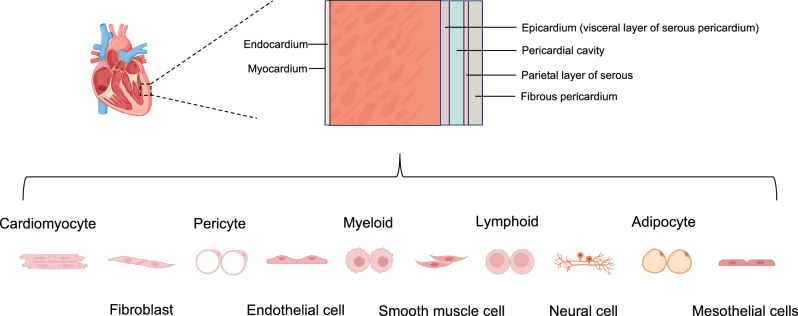
Composition of cardiac cell population. Part of the image was created with Biorender.com.

Heart regeneration involves not only cardiomyocyte proliferation but also the coordinated efforts of various cardiac cell populations within the cellular ecosystem of the injured heart. Locally, the potential for cardiac regeneration is determined by multiple highly interconnected processes, including cardiomyocyte proliferation, cardiac fibrosis, neovascularization, immune response, and energy metabolism^76^. The adult mammalian heart is composed of distinct tissues that contain niches of specialized cell types with site-specific functionality. Before the era of scRNA-seq, genetic tools and cellular markers were employed to quantify the most prominent cell types in the heart^77,78^. However, these approaches were constrained by the availability and specificity of cellular markers. Transcriptional profiling of cardiac cells, especially at the single-cell level through scRNA-seq and snRNA-seq^79,80^, has greatly informed our understanding of human cardiac cell heterogeneity (Fig. 3). Moreover, snRNA-seq data have provided new insights into the interactions between cardiomyocytes and adjacent cell types, such as endothelial cells, fibroblasts, mural cells (smooth muscle cells and pericytes), and immune cells, as well as the roles that these interactions play during heart regeneration.

### Cardiomyocytes

Cardiomyocytes are the primary functional cells of the heart. It is now well-accepted that proliferation of pre-existing cardiomyocytes is the major source of regenerated cardiac muscle, with adult mammalian cardiomyocyte cell cycle arrest and polyploidization identified as significant barriers to heart regeneration. For a broader discussion of the role of cardiomyocyte proliferation in heart regeneration, readers may refer to two of our previous reviews^20,81^.

Single-cell techniques offer the advantage of exploring the expression heterogeneity of the cardiomyocytes. Using scRNA-seq, Honkoop et al.^82^ uncovered that proliferating border zone cardiomyocytes in zebrafish have distinct transcriptomes compared to the non-proliferating remote cardiomyocytes, closely resembling embryonic cardiomyocytes. Moreover, these cells have reduced expression of mitochondrial genes and reduced mitochondrial activity, while glycolysis gene expression and glucose uptake are increased, indicative of metabolic reprogramming. Furthermore, the authors found that the metabolic reprogramming of border zone cardiomyocytes, induced by Nrg1/ErbB2 signaling, is crucial for their proliferation. This mechanism is conserved in murine hearts where cardiomyocyte proliferation is also induced by activating ErbB2 signaling.

In mammalian system, See et al. used snRNA-seq to analyze failing and non-failing adult hearts in both mouse and human, revealing subpopulations of cardiomyocytes with cardiac regeneration potential. This potential is supported by their capacity to dedifferentiate and upregulate cell cycle regulators after stress through lncRNAs^83^. Moreover, Cui et al.^84^ employed snRNA-seq analysis to map the dynamic transcriptional landscape of five distinct cardiomyocyte populations in healthy, injured, and regenerating neonatal mouse hearts. They identified immature cardiomyocytes that enter the cell cycle following injury and disappear as the heart loses its regenerative ability. The proliferative neonatal cardiomyocytes display a unique transcriptional program dependent on nuclear transcription factor Y subunit alpha and the nuclear factor erythroid 2-like 1 transcription factors, which exert proliferative and protective functions, respectively. Cardiac overexpression of these two factors conferred both proliferative and protective effects against ischemic injury in mature mouse hearts, which are otherwise non-regenerative. Farah et al.^85^ present human scRNA-seq and spatial transcriptomic data suggesting that cardiac fibroblasts may signal to cardiomyocytes during heart development, potentially inducing ventricular cardiomyocyte growth.

### Fibroblasts

Cardiac fibroblasts are one of the most abundant cell types in the heart, even though most of the cardiac mass is composed of cardiomyocytes. Cardiac fibroblasts are the major drivers of fibrosis, the mechanism of scar formation that prevents ventricular rupture after myocardial infarction. However, the fibrotic scar expansion and left ventricular dilation can eventually lead to heart failure. Interestingly, embryonic cardiac fibroblasts produce cytokines that can activate proliferation of cultured cardiomyocytes, and the structural proteins produced by cardiac fibroblasts may regulate cardiomyocyte cell cycle activity by modulating the stiffness of the extracellular matrix. This review mainly focused on the role of cardiac fibroblasts on cardiomyocyte proliferation. Readers may refer to a recent review by Chen et al.^86^ for a description of the general role of cardiac fibroblasts in the innate response to myocardial infarction, as well as their phenotypic plasticity and involvement in extracellular matrix production.

It has been shown that embryonic cardiac fibroblasts produce fibronectin and heparin-binding EGF-like growth factor, which activate proliferation in cultured mouse cardiomyocytes via β1-integrin signaling^87^. Moreover, periostin, which is primarily secreted by cardiac fibroblasts after myocardial infarction^88^, has been shown to induce cardiomyocyte cell cycle reentry and mitosis, and was associated with improved ventricular remodeling and myocardial function, reduced fibrosis and infarct size, and increased angiogenesis after myocardial infarction in mice^89^. In contrast, periostin deficiencies inhibited myocardial regeneration after myocardial infarction in neonatal mice^90^. More recently, Wang et al.^91^ integrated scRNA-seq data of mouse hearts at multiple postnatal stages and revealed switching of fibroblast subtypes from a neonatal to adult state which drives cardiomyocyte maturation. Furthermore, single-cell analysis of in vivo and in vitro cardiomyocyte maturation trajectories identifies highly conserved signaling pathways; pharmacological targeting of which substantially delays cardiomyocyte maturation in postnatal hearts, markedly enhances cardiomyocyte proliferation, and improves cardiac function in infarcted hearts.

### Endothelial cells

Endothelial cells, which line the inside of all blood and lymphatic vessels, play key roles in delivering oxygen and nutrients, regulating blood flow, modulating immune cell trafficking, and maintaining tissue homeostasis. Ma et al.^92^ established single‐cell transcriptome atlas of cardiac non‐cardiomyocytes in adult zebrafish heart and identified three major types of highly specialized endothelial cells—vascular endothelial cells, lymphatic endothelial cells, and endocardial endothelial cells.

#### Vascular endothelial cells

Vascular endothelial cells play a key role in responding to injury by rapidly and robustly revascularizing the injured area, which is required for efficient heart regeneration^93^. They migrate into the apical thrombus early after cardiac damage, develop into functional arteries, and precede cardiomyocyte ingrowth during mammalian heart regeneration^94^. Neonatal mouse hearts use a novel mechanism to build collateral arteries in response to injury. Arterial endothelial cells migrated away from arteries along existing capillaries and reassembled into collateral arteries, which termed “artery reassembly”. Artery endothelial cells expressed CXCR4, and following injury, capillary endothelial cells induced its ligand, CXCL12. CXCL12 or CXCR4 deletion impaired collateral artery formation and neonatal heart regeneration. Artery reassembly was nearly absent in adults but was induced by exogenous CXCL12^95^. Wang et al.^96^, performed single-cell transcriptome profiling of normal, failed, and partially recovered (left ventricular assist device treatment) adult human hearts and uncovered active engagement of ACKR1^+^-endothelial cells in regulating cardiomyocyte behavior. The injection of ACKR1^+^-endothelial cells preserved cardiac function after injury.

#### Lymphatic endothelial cells

In zebrafish, Harrison et al.^97^ found that the cardiac lymphatic system can influence the regenerative potential of the myocardium. Specifically, the lymphatic vasculature undergoes extensive lymphangiogenesis in response to cryoinjury in regenerating adult zebrafish hearts. A significant defect in scar reduction after cryoinjury is observed in zebrafish with impaired Vegfc/Vegfr3 signaling, which fail to develop intact cardiac lymphatic vessels.

In mammals, Vieira et al.^98^, reported that stimulation of cardiac lymphangiogenesis with VEGF-C in mice enhances the clearance of acute inflammatory response, thereby promoting cardiac repair after myocardial infarction. This effect is mediated by trafficking immune cells to draining mediastinal lymph nodes, a process dependent on lymphatic vessel endothelial hyaluronan receptor 1 (LYVE-1). Deletion of *Lyve1* in mice, which prevents the docking and transit of leukocytes through the lymphatic endothelium, leads to exacerbated chronic inflammation and long-term deterioration of cardiac function. Travisano et al.^99^ performed single nuclei multiomic analyses of human cardiac lymphatic endothelial cells and identified a population of cardiac lymphatic endothelial cells marked by the PROX1 and the lymphangiocrine RELN. This population was enriched in binding motifs of erythroblast transformation specific variant transcription factors. Moreover, Liu and colleagues reported that the reelin produced by lymphatic endothelial cells controls the proliferation of cardiomyocytes during heart development and improves neonatal cardiac regeneration^100^. However, another study with conflicting findings by Keller et al.^101^ found that blocking lymphangiogenesis through three different genetic approaches (pan-endothelial or lymphatic endothelial loss of the lymphangiogenic receptor VEGFR3, or global loss of the VEGF-C and VEGF-D ligands) did not impair left ventricular ejection fraction 2 weeks after myocardial infarction in mice.

### Smooth muscle cells and pericytes

Smooth muscle cells and pericytes, subtypes of mural cells, covers the myocardial capillaries in the human heart^102^. On average, a pericyte covers two or three endothelial cells in the heart^103^. Pericytes have been shown to improve cardiac function and enhance cardiac repair after myocardial ischemia via attenuation of cardiac remodeling, alleviation of inflammatory responses, and induction of angiogenesis^104^. Moreover, pericytes mediate neovascularization by releasing angiogenic factors in the injured area, which benefits cardiac recovery by providing additional nutrients and oxygen^105^. Some evidence also suggests that pericytes function as mesenchymal or progenitor cells in cardiovascular regeneration^104^. However, lineage tracing results from Quijada et al.^106^ indicate that pericytes do not exhibit multipotent progenitor cell characteristics following myocardial infarction. They did not observe protein marker expression representing each significant cardiac cell type (cardiomyocytes, resident fibroblasts, immune cells, or endothelial cells) in cardiac pericytes subjected to myocardial infarction. They further isolated mouse pericytes that had undergone sham or myocardial infarction surgery after 4, 7, or 14 days to represent the inflammatory, proliferative/reparative, and maturation phases of ischemic remodeling, respectively, and performed scRNA-seq analysis. The single-cell transcriptomic results indicated that in response to ischemic cardiac injury, pericytes expressed an overabundance of genes related to vascular permeability, proliferation, extracellular matrix (ECM) production, and fibrosis, peaking at post-injury day 7. This confirmed their role in vascular stability and suggested that pericytes contribute to fibrotic scar formation. Moreover, the transcriptional profile of pericytes supports experimental data showing mural cell separation from the vasculature following myocardial infarction and positioning at the site of injury 7 and 14 days post-injury. This process involves disruption and retraction from the basement membrane and subsequent vascular leakage. Consistently, another mouse lineage tracing and scRNA-seq study performed by Alex et al.^107^ demonstrated that in the infarcted region, a subpopulation of pericytes transiently expressed fibroblast markers. Pericyte-derived cells constituted ~4% of PDGFRα+ infarct fibroblasts during the proliferative phase of repair. These fibroblasts were overactive, expressing higher levels of ECM genes, integrins, matricellular proteins, and growth factors compared to fibroblasts from other sources. Another subset of pericytes contributed to infarct angiogenesis by forming a mural cell coat, stabilizing neovessels. ScRNA-seq showed that cells from pericyte lineage diversify after infarction, with increased expression of matrix genes and the emergence of a cluster with high expression of fibroblast markers. Trajectory analysis suggested that the diversification of infarct pericytes may be driven by proliferating cells. Furthermore, in vitro and in vivo studies identified TGF-β as a potential mediator in the fibrogenic activation of infarct pericytes. These findings demonstrate the critical significance of mural cells in cardiac repair.

### Immune cells

Accumulating evidence unequivocally demonstrates the contributions of the immune response to heart regeneration. Prior studies have found that both myeloid cells and lymphocytes are prominent immune cell populations within the healthy adult hearts of humans and mice^79,108^. Resident immune cells, including cardiac macrophages, are distributed throughout the heart, occupying spaces between cardiomyocytes in the epicardium, endocardium, valves, and nodal regions, thereby playing a role in maintaining organ homeostasis. Here, we focus on the role of cardiac-resident immune cells in heart regeneration. Myocardial infarction induces cardiac cell damage, leading to the release of various heterogenous molecules, including damage-associated molecular patterns and alarmins. Cardiac-resident macrophages and the endothelium detect these danger signals, initiating the recruitment of blood neutrophil to clear cellular debris from the tissue environment^109^.

Myeloid cells, consisting of macrophages, dendritic cells, and neutrophils etc., are the major population of cardiac-resident immune cells in humans. In a larval zebrafish cardiac injury model, Bruton et al.^110^ reported that macrophages activate the epicardium and induce cardiomyocyte proliferation. Mechanistically, macrophages are specifically recruited to the epicardial-myocardial niche, triggering the expansion of the epicardium. This expansion upregulates *vegfaa* expression, which in turn induces cardiomyocyte proliferation. Using genetic manipulation and single-cell transcriptomics, Apaydin et al.^111^ showed that receptor signaling activates an “extracellular matrix remodeling” transcriptional program in a subset of macrophages. This program plays as a key role in regulating matrix composition and turnover. Mechanistically, α-1-adrenergic receptor-activated macrophages activate collagen-12-expressing fibroblasts, which are crucial for the cardiac regenerative niche. This activation occurs through midkine-mediated paracrine signaling and promotes lymphatic and blood vessel growth as well as cardiomyocyte proliferation at the lesion site. Wei et al.^112^ conducted a comparative analysis using scRNA-seq to profile inflammatory cells derived from regenerative and non-regenerative hearts. They identified heterogeneous populations of macrophages and neutrophils, revealing alternative activation states and intercellular communication mechanisms that contribute to neutrophil retention and chronic inflammation. Within the macrophage population, two distinct subtypes were specifically enriched in regenerative hearts. These subtypes exhibited limited recovery following clodronate liposome treatment, which delays macrophage recruitment. To create a model devoid of resident macrophages without impeding the recruitment of circulating macrophages, the researchers administered clodronate liposome earlier, at 8 days before cryoinjury. Remarkably, zebrafish lacking resident macrophages still displayed deficiencies in revascularization, cardiomyocyte viability, debris clearance, and extracellular matrix remodeling/scar resolution, without compensatory support from circulating or monocyte-derived macrophages. These findings highlight the diverse functions of inflammatory cells and their intricate interactions, emphasizing the essential role of specific resident macrophages in facilitating zebrafish heart regeneration.

In mammals, Lavine et al.^113^ reported that the C-C chemokine receptor type 2 (CCR2)- macrophage pool orchestrates cardiomyocyte proliferation and neovascularization in neonatal hearts. Fate mapping and single-cell transcriptomics identified a specific subset of embryonic CCR2- cells that were capable of self-renewal with negligible monocyte input under steady state, but these cells were nearly abolished in infarcted adult myocardium. Moreover, Reboll et al.^114^ reported that the monocyte- and macrophage-derived cytokine METRNL (meteorin-like) drives post-infarction angiogenesis and acts as a high-affinity ligand for the stem cell factor receptor KIT (KIT receptor tyrosine kinase). In a mouse model of myocardial infarction, METRNL promoted infarct repair by selectively expanding the KIT-expressing endothelial cell population in the infarct border zone. Moreover, Metrnl-deficient mice failed to mount this KIT-dependent angiogenic response and developed severe post-infarction heart failure.

Cardiac lymphoid cells, primarily composed of T cells, B cells, and NK cells, are less abundant than myeloid cells in the heart. Weirather et al.^115^ reported that Treg cells, critical mediators of immune suppression, promote healing of the infarcted area by inducing macrophage differentiation toward an anti-inflammatory phenotype. Comparison of CD4^+^ T cells across different developmental stages revealed an intrinsic mechanism that makes CD4^+^ T cells more prone to differentiating into Treg cells upon T-cell receptor stimulation in neonatal mice. This property diminishes after the first two postnatal weeks^116^.

### Neuronal cells

The brain is the central regulatory authority of the cardiovascular system; however, cardiac neuronal cells also exert influence. Single-cell transcriptomic analysis revealed the presence of neuronal cells in various anatomical positions of the heart, constituting approximately 1% of cells in the adult human heart. The neurons have been shown to exert a significant influence on cardiac functionality, participating in the modulation of cardiac rhythmicity and affecting heart regeneration capacity^67^.

### Adipocytes

Adipocytes, as their name suggests, constitute adipose tissues. Epicardial adipose tissue is metabolically active and lies between myocardium and visceral layer of pericardium^117^. Recent studies emphasized the benefits of cardiac adipose tissue in heart remodeling, such as reducing infarct size, enhancing neovascularization, and regulating immune response, through a series of cellular mechanisms. However, the direct differentiation capacity of cardiac adipose-derived stem cells into cardiomyocytes has been suspected due to the absence of long-term cell retention post transplantation. It was revealed that most labeled adipocytes disappeared 28 days after being transplanted into the heart^118^. On the other hand, cumulative evidence strongly suggests that the paracrine effect accounted for cardiac repair. However, it remains challenging to pinpoint the specific role of cardiac adipose tissue, as many adipocytokines are derived from multiple tissues. Further studies are highly anticipated to elucidate the pivotal mechanisms involved.

### Endocardial cells

Endocardial cells have emerged as a new player in cardiac repair and regeneration. Endocardial cells lining the heart lumen are coronary vessel progenitors during embryogenesis. Re-igniting this developmental process in adults could regenerate blood vessels lost due to cardiac injury. D’Amato et al.^119^ used mouse genetics and scRNA-seq to identify regulators of endocardial angiogenesis. By integrating scRNA-seq data of endocardial-derived coronary vessels from mid- and late-gestation, they identified a Bmp2-expressing transitioning population specific to mid-gestation and discovered that Bmp2 overexpression after birth reactivates endocardial angiogenesis in neonatal mouse models of myocardial infarction.

### Epicardial cells

The epicardium is a layer of mesothelial tissue that envelops the heart in all vertebrates. Epicardial activity appears to be an important element of heart regeneration. On one hand, an active epicardium plays a substantial role in successful cardiac regeneration in adult zebrafish, newts, and developing mammalian systems. On the other hand, the epicardium is reported to be quiescent in adult mammalian and human hearts, which lack regenerative capabilities.

In zebrafish, regeneration proceeds through two coordinated stages following the resection of the ventricular apex. First, cardiac progenitor cells are detected by cardiogenic markers. Second, epicardial tissue surrounding both cardiac chambers induces developmental markers and rapidly expands, creating a new epithelial cover for the exposed myocardium. A subpopulation of these epicardial cells undergoes epithelial-to-mesenchymal transition, invades the wound, and provides new vasculature to regenerating muscle^120^.

In humans, Bargehr et al.^121^ reported that cotransplantation of human embryonic stem-cell-derived epicardial cells and cardiomyocytes doubled the proliferation rates of graft cardiomyocytes in vivo. This resulted in a 2.6-fold increase in cardiac graft size and simultaneously enhanced both graft and host vascularization. Notably, cotransplantation improved systolic function compared to hearts receiving either cardiomyocytes alone, epicardial cells alone, or a vehicle. Knight-Schrijver et al.^122^ combined fetal and adult human hearts using scRNA-seq and snRNA-seq techniques to compare epicardial cells from both stages. The authors identified a migratory fibroblast-like epicardial population exclusively in the fetal heart and fetal epicardium, which expressed angiogenic gene programs, while the adult epicardium was primarily mesothelial and immune responsive. Furthermore, the authors predicted that adult hearts might still receive paracrine signals from fetal epicardial cells, including WNT signaling with the endocardium, reinforcing the validity of regenerative strategies that involve administering or reactivating epicardial cells in situ. Finally, the authors explained the graft efficacy of human embryonic stem-cell-derived epicardium model reported by Bargehr et al.^121^ by noting its similarity to the human fetal epicardium. Another group also identified a cell population induced by regeneration within epicardium-derived cells and elucidated Angiopoietin 4 (Angpt4) as a specific regulator of heart regeneration^123^. Expression of Angpt4 is selectively and temporarily upregulated in regeneration-induced cells, initiating a signaling cascade from epicardium-derived cells to endocardium via the Tie2-MAPK pathway, subsequently activating cathepsin K in cardiomyocytes through retinoic acid signaling. Depletion of angpt4 results in deficiencies in scar tissue resolution and cardiomyocyte proliferation, while overexpression of angpt4 expedites the regeneration process. Additionally, the study revealed that ANGPT4 promotes the proliferation of neonatal rat cardiomyocytes and enhances cardiac repair in mice following myocardial infarction, indicating its conserved function across mammals.

### Cardiac cellular communication

Li et al.^124^ used scRNA-seq, snRNA-seq, and spatial transcriptomics to interrogate the microenvironment and to probe cell-to-cell communication within the injured mouse heart. The researchers analyzed neonatal mouse hearts alongside normal adult mouse hearts, adult mouse hearts with heightened activity of YAP (YAP5SA), and adult human hearts post-heart attack. This comprehensive approach across different species revealed common cellular patterns localized in specific ‘niches,’ potentially indicating a state conducive to regeneration. Initially focusing on specific subsets of cardiomyocytes, they identified a cluster labeled aCM2 present in YAP5SA-expressing adult and neonatal hearts but absent in non-regenerative adult mouse hearts. However, examination of existing snRNA-seq data and newly collected spatial transcriptomics data in human hearts unveiled the presence of aCM2 in the border zone of injury after a heart attack, suggesting the involvement of other cell types in establishing a conducive environment for heart muscle regeneration. The researchers further identified distinct subsets of cardiac fibroblasts and macrophages localized in specific niches that were significantly more abundant in neonatal and YAP5SA adult hearts compared to injured adult mouse or human hearts. They propose that these cell types may communicate with aCM2 and facilitate heart muscle regeneration, rather than hypertrophy, thus forming a pro-regenerative cellular triad in the border zone.

### Extracellular microenvironment

The cardiac ECM was once believed to function primarily as an inert scaffold, but it is now recognized as a dynamic and adaptable microenvironment that plays a crucial role in cardiac function and regeneration. The mammalian cardiac ECM consists of structural components such as collagens, fibronectin, and heparin-binding epidermal growth factor-like growth factor. It has been reported that the stiffness of the local microenvironment crucially determines the heart’s ability to regenerate. Cardiac regenerative capacity can be restored in neonatal mice by pharmacologically reducing stiffness^125^. Furthermore, Bassat et al.^126^ reported that Agrin, a component of neonatal extracellular matrix, is required for the full regenerative capacity of neonatal mouse hearts. Recombinant Agrin promotes the division of cardiomyocytes derived from mouse and human induced pluripotent stem cells through a mechanism that involves the disassembly of the dystrophin-glycoprotein complex, and Yap- and ERK-mediated signaling. A single administration of Agrin promotes cardiac regeneration in adult mice after myocardial infarction. In pigs, a single dose of recombinant human Agrin improved heart function, reduced infarct size and fibrosis, and mitigated adverse remodeling parameters 28 days after myocardial infarction^127^. Short-term myocardial infarction experiments, along with complementary murine studies, revealed that Agrin’s mechanisms of action include myocardial protection, improved angiogenesis, inflammatory suppression, and cell cycle reentry.

## Conclusions and future perspectives

In conclusion, ischemic heart disease represents a significant global health burden, with myocardial infarction-induced cardiomyocyte loss contributing substantially to mortality rates. While the regenerative capacity of the adult mammalian heart is limited, recent research has expanded our understanding of heart regeneration beyond mere cardiomyocyte proliferation. This review emphasizes the importance of adopting a systematic approach to studying heart regeneration, which encompasses not only the organisms themselves but also their surrounding environments. By examining heart regeneration at a cellular resolution and exploring the intricate cellular crosstalk among diverse cardiac populations, we can gain a comprehensive understanding of the underlying processes.

This holistic perspective offers valuable insights into heart regeneration and holds promise for the development of innovative therapeutic strategies. Future research endeavors should focus on elucidating the intricate mechanisms that govern heart regeneration, including the roles of various cell types, signaling pathways, and environmental factors. With the advancements of spatial transcriptomics^85^, snATAC-seq^128,129^ and single-cell multi-modal technologies and analyses, there can not only be a more expansive survey of the physiology, but also a deeper dive into the finer mechanisms of heart regeneration. Furthermore, continued efforts are needed to translate these findings into effective clinical interventions aimed at combating ischemic heart disease.

In the future, interdisciplinary collaborations between researchers from fields such as cardiology, developmental biology, tissue engineering, and regenerative medicine will be crucial for advancing our knowledge of heart regeneration and translating it into clinical practice. By harnessing the power of cutting-edge technologies and approaches, we can pioneer the development of novel regenerative therapies that have the potential to revolutionize the treatment of ischemic heart disease and improve patient outcomes.
